# Complete Genome Sequences of Papillomavirus Isolates from the Oral Cavity, Skin, and Feces of Wild Rats

**DOI:** 10.1128/MRA.01258-18

**Published:** 2018-11-01

**Authors:** Yu Ling, Fei Mo, Yumin He, Min Zhao, Hui Xu, Wen Zhang

**Affiliations:** a Department of Microbiology, School of Medicine, Jiangsu University, Zhenjiang, Jiangsu, China; b The Affiliated Hospital of Jiangsu University, Zhenjiang, Jiangsu, China; Loyola University Chicago

## Abstract

Six genome sequences of papillomavirus were determined from oral and skin swabs and fecal samples collected from wild rats. Three genomes were 7,722 bp, two genomes were 7,716 bp, and one was 7,730 bp, displaying typical papillomavirus genome organizations. Phylogenetic analysis revealed that these six genomes belonged to two different clusters.

## Abstract (precis)

Six genome sequences of papillomavirus were determined from oral and skin swabs and fecal samples collected from wild rats. Three genomes were 7,722 bp, two genomes were 7,716 bp, and one was 7,730 bp, displaying typical papillomavirus genome organizations.

## ANNOUNCEMENT

Papillomaviruses (PVs) are known to infect a wide variety of mammals, birds, and reptiles (1) and are classified into genera based on the open reading frame (ORF) L1 (2). While the overwhelming majority of PVs only infect the epithelium (2, 3), the bovine papillomaviruses have the ability to infect both epithelial and mesenchymal cells and to infect multiple species (4). PVs are believed to have coevolved with their vertebrate host species, a hypothesis supported by the fact that PVs of closely related host species are generally closely related themselves (5, 6). Recently, more complete genomes of PVs were reported in nonhuman species (7–14).

Here, using viral metagenomics, we investigated the viral nucleic acid sequences from oral swabs, skin swabs, and feces. All samples were from 20 wild rats collected in Zhenjiang City, China, in 2016. Viral metagenomic analysis was performed as previously reported (15, 16). Briefly, 60 sample supernatants were pooled into 5-sample pools according to the 3 sample types and generated 12 pools where each type of sample included 4 pools. Total nucleic acid was then isolated using the QIAamp MinElute virus spin kit (Qiagen) according to manufacturer’s protocol. Twelve libraries were then constructed using the Nextera XT DNA sample preparation kit (Illumina) and sequenced using the MiSeq Illumina platform with 250-bp paired-end reads with dual barcoding for each pool. The total numbers of sequence reads generated for the 12 libraries were 3,204,222 (feces01), 2,009,444 (feces02), 2,330,836 (feces03), 1,674,810 (feces04), 203,916 (oral01), 212,296 (oral02), 125,674 (oral03), 39,314 (oral04), 292,992 (skin01), 87,252 (skin02), 81,526 (skin03), and 61,134 (skin04). An in-house analysis pipeline running on a 32-node Linux cluster was used to process the data. Reads were considered duplicates if bases 5 to 55 were identical and only one random copy of duplicates was kept. Clonal reads were removed, and low sequencing quality tails were trimmed using Phred quality score 10 as the threshold. Adaptors were trimmed using the default parameters of VecScreen, which is NCBI BLASTn with specialized parameters designed for adapter removal. The cleaned reads from each library were *de novo* assembled within each barcode using the Ensemble assembler (17). Contigs and singlets reads were then matched against a customized viral proteome database using BLASTx with an E value cutoff of <10^−5^.

We acquired six complete genomes by assembling the viral reads showing significant similarity to papillomavirus and conducting common PCR bridging the gaps between contigs using primers designed based on their closest relative genomes in GenBank, which are hereafter referred to as Ne-or02-zj, Or01-zj, Or02-zj, Sk01-zj, Sk02-zj, and Fe02-zj. Ne-or02-zj was found in the oral swab sample (containing 434 reads in library oral02) and shared 86.25% nucleotide sequence identity with a papillomavirus strain (GenBank accession no. KY370097) over the complete genome based on a BLASTn search, which was detected in Rattus norvegicus according to the sequence annotation in GenBank. Or01-zj (containing 25,285 reads in library oral01) and Or02-zj (containing 1,402 reads in library oral02) were detected in two different oral swab pools. Sk01-zj (containing 3,187 reads in library skin01) and Sk02-zj (containing 1,393 reads in library skin02) were found in two different skin swab sample pools. Fe02-zj (containing 81 reads in library feces02) was acquired from a fecal sample pool. A BLASTn search in GenBank based on the complete genome sequences of Or01-zj, Or02-zj, Fe02-zj, Sk01-zj, and Sk02-zj showed their best match was Rattus norvegicus papillomavirus 2 (GenBank accession no. HQ625441) (18). Five distinct ORFs on the same coding strand in the genomes of Or01-zj, Or02-zj, Fe02-zj, Sk01-zj, and Sk02-zj were identified using Geneious version 11.0, including the early genes E6, E1, and E2 and the late genes L2 and L1, while there were six ORFs in the genome of Ne-or02-zj which had an additional E7 gene before E6.

**FIG 1:**
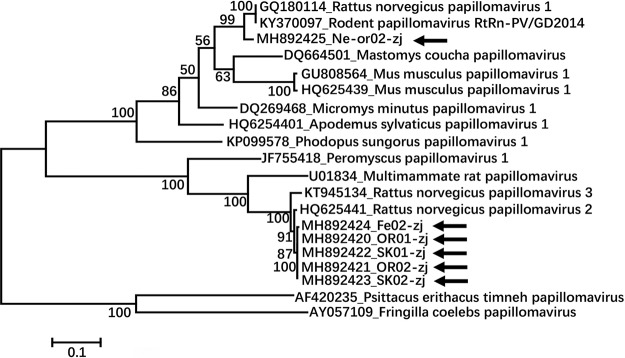
Phylogenetic analysis based on amino acid sequence alignment of the complete L1 protein. The sequences in the phylogenetic tree included the 6 papillomaviruses identified in this study, 12 papillomaviruses with complete genomes from rodents available in GenBank, and 2 complete papillomavirus genomes from birds as an outgroup. The GenBank number of the referenced genomes are provided. The six papillomavirus strains identified in this study are marked by arrows.

To determine the divergence in sequence between the PVs found here and other PVs, amino acid sequence alignment of the complete L1 protein was performed using Clustal W version 2.0 (19), and a phylogenetic tree was generated using the maximum-likelihood method in MEGA7 (20) with 1,000 bootstrap resamples of the alignment data sets. Phylogenetic analysis indicated that the six papillomavirus genomes fell into two different clusters, where Ne-or02-zj clustered with Rodent papillomavirus isolate RtRn-PV/GF2014 (GenBank accession no. KY370097), and the other five strains clustered with R. norvegicus papillomavirus 2 (GenBank accession no. HQ625441) (Fig. 1).

### Data availability.

The raw sequence reads were deposited in the Sequence Read Archive under accession no. SRX4791096 to SRX4791099, SRX4791100, SRX4791137, SRX4791138, SRX4791154, SRX4791673, SRX4791804, SRX4791859, and SRX4791861. The six genomic sequences were deposited in GenBank under accession no. MH892420 to MH892425.
